# Using a literary and arts magazine to promote mental health and wellness among trainee healthcare professionals: lessons from a Canadian student-led project

**DOI:** 10.1192/bji.2023.40

**Published:** 2024-05

**Authors:** Carl Zhou, Keerthana Pasumarthi, Isabella Liang, Jim Xie, Andrew Toyin Olagunju

**Affiliations:** 1 MD, Resident Physician, Department of Psychiatry, University of Ottawa, Ottawa, Canada; 2 MD, Resident Physician, Department of Medicine, University of Toronto, Toronto, Canada; 3 MD, Resident Physician, Department of Family Medicine, McMaster University, Hamilton, Canada; 4 BHSc, Medical Student, Michael G. Degroote School of Medicine, McMaster University, Hamilton, Canada; 5 MBBS, MSc, PhD, FWACP, FMCPsych., Assistant Professor, Department of Psychiatry and Behavioral Neurosciences, McMaster University/St. Joseph's Healthcare Hamilton, Hamilton, Canada

**Keywords:** Artistic expression, trainee healthcare professional, mental health, student-led project, wellness

## Abstract

*Breathe* is a student-led literary and arts magazine whose goal is to provide a platform for creative expression about mental health issues and promote mental wellness among trainee healthcare professionals using student-submitted art and written pieces. Select pieces were published to improve readers’ understanding of and self-reflection on mental health. Common themes among the submissions include life outside of healthcare, imposter syndrome and coping with stress. This novel project had high satisfaction reported by 87.5% of surveyed readers. We advocate for improved mental health awareness and increased use of artistic expression as a coping strategy against stressors in healthcare education worldwide.

The emotional wellness of trainee healthcare professionals is recognised as an important public health issue.^1^ Trainees are exposed to a unique set of stressors, including demanding workloads, high stakes and emotionally taxing patient encounters.^2^ Consequently, the rates of psychological distress, burnout and other stress-related mental health conditions are significantly higher in this group than in the general population.^2^ Such conditions not only compromise overall well-being but are also associated with increased medical errors and patient dissatisfaction.^3^

These alarming findings have prompted academic institutions to include various wellness promotion content in the academic curriculum.^1,2^ Although these efforts are laudable, trainees can feel uncomfortable with structured wellness activities that are embedded in the curriculum, as individual definitions of mental wellness are heterogeneous.^4^ Thus, there is a preference among trainees for student-led wellness activities, which tend to emphasise a diverse conceptualisation of mental wellness.^4^ In this light, student-led wellness activities are one of many tools that can facilitate healthy coping via the safe discussion and normalisation of mental health struggles. Ultimately, further research and system-level change are needed to address both the mental wellness crisis and the discomfort with formal wellness interventions among trainees.^5^

Struggles with stress, burnout and psychological distress among trainees were particularly intensified globally during the COVID-19 pandemic.^6^ Sadly, mental health stigma in the healthcare field remains a prevalent issue and poses a significant barrier to treatment and recovery for individuals with mental illness.^2^ The paucity of mental health-related platforms for self-expression in healthcare education inspired a group of trainees (including C.Z., K.P., I.L. and J.X.) to develop an outlet for their peers to share personal experiences, reflections and strategies to promote wellness in the form of an online literary and arts magazine *Breathe* (www.thebreathemag.com). The magazine's ultimate goal is to destigmatise mental health struggles and serve as a coping strategy against the various system- and work-related causes of distress for trainee healthcare professionals.

To accomplish these goals, the artistic medium was chosen for its subjectivity, unstructured nature and potential therapeutic effects. In the medical context, art and literature therapy are forms of psychotherapy that use creative expression to promote mental wellness.^7^ These therapies are based on the idea that the creative process can be therapeutic, allowing individuals to express and process their thoughts, feelings and emotions in a safe and non-threatening environment.^7^ Exposure to art or participation in group-based creative writing workshops can improve anxiety and depressive symptoms and be protective against burnout.^8,9^

## Project description

*Breathe* is a free-to-read, student-led online literary and arts magazine created by a team of trainee healthcare professionals at McMaster University in Canada. The target audience is all trainee healthcare professionals across Canada. The magazine showcases literary and artistic expressions about mental health and wellness during healthcare education. Some common themes among the submissions include pre-existing mental illness and its exacerbation, difficult experiences during training (e.g. loss of a patient, stress, imposter syndrome) and successful coping strategies used by trainees. The magazine includes visual art such as paintings, digital art, drawings and photography, as well as written pieces such as short stories, poetry and reflections. Interviews featuring individuals with profound mental health experiences in healthcare are also included. Supplementary Fig. 1, available at https://dx.doi.org/10.1192/bji.2023.40, outlines the steps involved in the magazine's development.

## Post-publication evaluation

This study to evaluate the potential benefits of *Breathe* magazine received ethical approval from the Hamilton Integrated Research Ethics Board (project ID: 16578). Implied informed consent through survey participation was obtained from all participants in compliance with the Research Ethics Board's requirements. After the first magazine issue was published, readers were emailed a request to complete an anonymised online survey assessing their satisfaction and knowledge acquisition using a 5-point Likert scale. Their contact information was obtained via the magazine website, which had requested email registration prior to viewing.

As shown in Table 1, descriptive statistics were used to analyse the survey data of 56 respondents out of 174 readers contacted (32.2% response rate). Overall, 85.7% of respondents reported that *Breathe* had provided them with an effective coping strategy for stress, 82.2% would read a future issue and 87.5% would recommend *Breathe* to others. Moreover, 69.6% of respondents agreed that their mental wellness (referred to as ‘well-being' in the wording of the survey) improved after reading the magazine, 91.1% of respondents indicated interest in using artistic media to cope with stress and 96.4% of respondents felt more comfortable with discussing mental health topics in the future. Overall, 87.5% of respondents were satisfied with *Breathe*.

**Table 1:** Reader responses to post-publication survey using a 5-point Likert scale (*n* = 56)

Questions	Strongly agree, *n* (%)	Agree, *n* (%)	Neutral, *n* (%)	Disagree, *n* (%)	Strongly disagree, *n* (%)	Mean score (s.d.)
*Breathe* magazine provided me with an effective coping strategy for stress	16 (28.6)	32 (57.1)	7 (12.5)	1 (1.79)	0	4.13 (0.68)
I am interested in reading future issues of *Breathe* magazine	22 (39.3)	24 (42.9)	9 (16.1)	1 (1.79)	0	4.20 (0.77)
I would recommend this magazine to others	22 (39.3)	27 (48.2)	7 (12.5)	0	0	4.27 (0.67)
I experienced improvements in my mental well-being after reading the magazine	14 (25.0)	25 (44.6)	15 (26.8)	2 (3.57)	0	3.91 (0.81)
I am interested in using artistic media such as visual art or writing to cope with stress in the future	21 (37.5)	30 (53.6)	4 (7.14)	1 (1.79)	0	4.27 (0.67)
*Breathe* magazine improved my comfort with discussing mental health in the healthcare field	20 (35.7)	34 (60.7)	2 (3.57)	0	0	4.32 (0.54)
Overall, I feel satisfied with my experience of reading *Breathe* magazine	16 (28.6)	33 (58.9)	6 (1.07)	1 (1.79)	0	4.14 (0.67)

## Discussion

### Reflections and recommendations

The launching of this novel project presented unique challenges. We offer some recommendations for colleagues who are interested in pursuing similar projects.

Considerable effort should be made to market the project prior to publication, for the purposes of securing future readership and artistic submissions. Social media should serve as the primary marketing channel, owing to its accessibility and low cost, but we also had success by directly contacting academic institutions and adjacent stakeholders for promotional and financial support. Having multiple means of promotion may help to achieve a more diverse pool of readers and submissions.

The project's vision and style should be made clear to the team prior to the commencement of work. We found frequent meetings and the employment of a team-wide ‘mood board’, where designers could compile images that convey general feelings they wished to express in the final publication, to be helpful for maintaining a unified project aesthetic. Moreover, if there are several subteams focused on different aspects of the project, difficulties might arise in keeping the entire team updated on progress. Employing more precise communication methods, being transparent about deadlines and implementing frequent check-ins to assess progress by the leaders can be helpful.

There should be clear plans for succession. Because the project is student-led, team turnover is expected as members graduate from their training. Recruitment should begin soon after the project's publication to take advantage of the surge in readership. General recruitment should take place through existing marketing channels, but current team members should be encouraged to participate in leadership roles, given their experience. Around the same time, the anticipated dates for future publications should be announced so that the audience's interest does not wane. Regular communication via marketing channels can help to maintain interest.

Finally, project improvement should be considered following publication, preferably via anonymous online surveys, given the sensitive nature of mental health issues. We suggest collecting useful demographic data such as academic programme, location of training and self-disclosure of reader and contributor status. The precise number of unique readers should also be collected via (a) website analytics or (b) mandatory email registration to view the magazine. The former might incur additional cost, whereas the latter might deter readership. For the inaugural issue, we asked readers to register by email prior to viewing, but this was not mandatory for accessing the magazine. Therefore, our email list of 174 readers likely does not reflect actual readership.

### Future directions

The inaugural issue of *Breathe* magazine received high satisfaction ratings from its readers. New issues of the magazine will be released annually, followed by post-publication surveys for programme improvement purposes. Limitations of the inaugural survey include a low response rate and imperfect data collection methods, as outlined above. Future work should use validated tools to assess well-being or wellness (e.g. the General Well-Being Schedule^10^) to be more methodologically rigorous. There is an ongoing effort to further disseminate the magazine and increase its accessibility across Canada. Similar projects can be implemented in other languages, at other academic institutions and targeting other geographic locations. However, student-led projects are inherently hampered by the lack of experience that students have in project management and creation. Although *Breathe* magazine and other similar projects continue to tackle the challenges described in this article, official support from academic institutions and professional organisations would probably amplify their impact. This support might include promotional aid, sponsorship, endorsement or ownership, and support for editorial team members to encourage their work (e.g. official acknowledgement, academic credit).

## Supporting information

Zhou et al. supplementary material Zhou et al. supplementary material

## Data Availability

The data that support the findings of this study are available from the corresponding author on reasonable request.
