# Using a literary and arts magazine to promote mental health and wellness among trainee healthcare professionals: lessons from a Canadian student-led project – ADDENDUM

**DOI:** 10.1192/bji.2024.10

**Published:** 2024-05

**Authors:** Carl Zhou, Keerthana Pasumarthi, Isabella Liang, Jim Xie, Andrew Toyin Olagunju

This article was originally published with the declaration of interest omitted. The declaration of interest has since been added and this addendum prepared.
