# Supplementary material for: Using a literary and arts magazine to promote mental health and wellness among trainee healthcare professionals: lessons from a Canadian student-led project
Source: BJPsych Int. 2024 May;21(2):32–4. doi: 10.1192/bji.2023.40 (PMC11035964; doi:10.1192/bji.2023.40)
Supplement: Zhou et al. supplementary material [file S2056474023000405sup001.docx]

**Figure 1.** A flow diagram depicting the approaches and steps involved in creating *Breathe Magazine*.

**Initiation**

1. Researched and contacted current literary and arts magazines in the healthcare field
2. Determined scope and audience of project

**Defining the Problem**

1. Conducted literature search to identify existing initiatives in the healthcare field
2. Reflected on experiences and communicated with peers to discern specific adversities

**Team Recruitment and Stakeholder Consultation**

1. Recruited team members for content curation, editing, and graphic design
2. Consulted stakeholders to guide and support the project

**Product and Dissemination**

1. Established social media and online presence
2. Opened submissions and advertised via email lists and social media
3. Received and edited submissions
4. Made call for interviews
5. Established visual theme and prepared magazine for publication and print
6. Contacted stakeholders for support and guidance regarding dissemination of final product
7. Published final product online and advertised via email lists and social media
